# Ipsen 5i is a Novel Potent Pharmacoperone for Intracellularly Retained Melanocortin-4 Receptor Mutants

**DOI:** 10.3389/fendo.2014.00131

**Published:** 2014-08-04

**Authors:** Ya-Xiong Tao, Hui Huang

**Affiliations:** ^1^Department of Anatomy, Physiology and Pharmacology, College of Veterinary Medicine, Auburn University, Auburn, AL, USA

**Keywords:** G protein-coupled receptor, melanocortin-4 receptor, obesity, pharmacological chaperone/pharmacoperone, protein misfolding

## Abstract

Inactivating mutations of the melanocortin-4 receptor (*MC4R*) cause early-onset severe obesity in humans. Comprehensive functional studies show that most of the inactivating mutants of the MC4R are retained intracellularly. In the present study, we investigated whether a small molecule inverse agonist of the MC4R, Ipsen 5i, could act as a pharmacoperone and correct the cell surface expression and function of intracellularly retained mutant MC4Rs using multiple cell lines, including HEK293 and two neuronal cell lines. We showed that Ipsen 5i rescued the cell surface expression of all 11 intracellularly retained mutant MC4Rs studied herein in at least one cell line. Ipsen 5i functionally rescued seven mutants in all cell lines used. One mutant (Y157S) was functionally rescued in HEK293 cells but not in the two neuronal cell lines. Ipsen 5i increased cell surface expression of three mutants (S58C, G98R, and F261S) but did not affect signaling. Ipsen 5i had no effect on mutant MC4Rs with other defects (Δ88-92, D90N, I102S) or no defect (N274S). It also did not affect trafficking of a misrouted MC3R mutant (I335S). Cell impermeable peptide ligands of the MC4R or cell permeable small molecule ligand of δ opioid receptor could not rescue misrouted mutant MC4R. In summary, we demonstrated that Ipsen 5i was a novel potent pharmacoperone of the MC4R, correcting trafficking and signaling of a significant portion (73%) of intracellularly retained mutants. Additional studies are needed to demonstrate its *in vivo* efficacy.

## Introduction

The melanocortin-4 receptor (MC4R) is a G protein-coupled receptor (GPCR) that is widely expressed in the central nervous system including the cortex, thalamus, hypothalamus, hippocampus, brainstem, and spinal cord (1, 2). The MC4R plays a vital role in the leptin-melanocortin pathway in regulating energy homeostasis, affecting both energy intake and expenditure (3, 4). Tissue-specific knockout studies revealed that the MC4R expressed in the paraventricular nucleus and/or amygdala neurons regulates food intake (5) whereas the MC4R expressed in the cholinergic neurons regulates energy expenditure and hepatic glucose production (6). Inactivating mutations of the *MC4R* cause early-onset severe obesity (7–9), which is the most common monogenic form of obesity in humans (10).

Most of the inactivating MC4R mutants are misfolded and trapped intracellularly by the stringent endoplasmic reticulum (ER) quality control system (11–13). These mutant MC4Rs may only have minor folding defect but retain pharmacological function. If they are escorted onto the cell surface, they can potentially bind the ligand and initiate signaling. Several studies have attempted to promote the anterograde trafficking of these mutant MC4Rs, using molecular chaperone (14), chemical chaperone (15), or pharmacological chaperone (or pharmacoperone) (13, 16–18).

The therapeutic potential of molecular and chemical chaperones is limited due to disruption of proteostasis or significant side effects whereas pharmacoperone is a promising approach. It has been tested in numerous human diseases caused by misfolded proteins, including neurodegenerative diseases, cystic fibrosis, lysosomal storage diseases, and cancer, with several promising clinical trials underway [reviewed in Ref. (19–21)]. Misfolding is also the most common defect in diseases caused by mutations in GPCR genes (22). Pharmacoperones have also been identified for several GPCRs, including rhodopsin, V2 arginine vasopressin receptor, gonadotropin-releasing hormone receptor, calcium-sensing receptor, and others [reviewed in Ref. (21)]. In the MC4R, we and others reported a few molecules that act as pharmacoperones (13, 16–18). These compounds have low affinities for the MC4R, therefore, usually need high concentrations (10^−6^ M and higher) to achieve any rescue.

Ipsen 5i was synthesized and identified as a high-affinity antagonist and partial inverse agonist of MC4R competing with [Nle^4^,D-Phe^7^]-α-melanocyte stimulating hormone (NDP-MSH) for binding to the MC4R (23–25). We reported recently that although it decreases basal signaling at the classical Gs-cAMP pathway, it acts as an agonist in the mitogen-activated protein kinase pathway (26). In this study, we investigated whether Ipsen 5i could act as a pharmacoperone promoting the proper folding and trafficking of intracellularly retained mutant MC4Rs using multiple cell lines. A total of 15 mutants were studied, including 11 (S58C, N62S, I69R, P78L, C84R, G98R, Y157S, W174C, P260Q, F261S, and C271Y) that are retained intracellularly and four (Δ88-92, D90N, I102S, and N274S) that are expressed relatively normally at the cell surface with other or no defects.

## Materials and Methods

### Materials and plasmids

NDP-MSH was purchased from Peptides International (Louisville, KY), α-MSH from Pi Proteomics (Huntsville, AL, USA), SHU9119 from Tocris Bioscience (Ellisville, MO, USA), and naltrexone hydrochloride from Alfa Aesar (Ward Hill, MA, USA). Ipsen 5i was custom synthesized by Enzo Life Science (Plymouth Meeting, PA, USA). [^125^I]-cAMP was iodinated with chloramine T method (25). Wild type (WT) and mutant human MC4R with N-terminal c-myc tag and human melanocortin-3 receptor (MC3R) with N-terminal 3 × HA tag were previously constructed and sequenced (16, 27–30).

### Cell culture and transfection

Human embryonic kidney (HEK) 293, Neuro2a, and N1E-115 cells were purchased from American Type Culture Collection (Manassas, VA, USA) and cultured in Dulbecco’s modified Eagle’s medium supplemented with 10% newborn calf serum (HEK293 cells) or 10% fetal bovine serum (Neuro2a and N1E-115 cells) at 37°C. HEK293 cells were stably transfected using calcium phosphate precipitation method for transfection and 0.2 mg/ml G418 for selection. Neuro2a and N1E-115 cells were transiently transfected using jetPRIME transfection reagent (Polyplus-transfection, New York, NY, USA) and approximately 24 h later were used for ligand treatment. Cells were treated with indicated concentrations of ligands or 0.1% dimethyl sulfoxide (DMSO) as control for 24 h at 37°C. All cell culture plates were pretreated with 0.1% gelatin before cell plating unless noted otherwise.

### Confocal microscopy

HEK293 stable cells seeded into poly-d-lysine-coated 8-well slides (Biocoat Cellware from Falcon, B&D Systems, Franklin Lakes, NJ, USA) were treated with 0.1% DMSO or 10^−6^ M Ipsen 5i for 24 h. On the day of experiment, cells were washed with phosphate buffered saline for immunohistochemistry (PBS-IH, 137 mM NaCl, 2.7 mM KCl, 1.4 mM KH_2_PO_4_, 4.3 mM Na_2_HPO_4_, pH 7.4) and fixed with 4% paraformaldehyde for 15 min. After blocking with 5% bovine serum albumin (BSA) in PBS-IH for 1 h, cells were incubated with mouse anti-myc 9E10 monoclonal antibody (Developmental Studies Hybridoma Bank, The University of Iowa, Iowa City, IA, USA) 1:40 diluted in PBS-IH containing 0.5% BSA for 1 h. Cells were then washed and incubated with Alexa Fluor 488-labeled goat anti-mouse antibody (Invitrogen, Grand Island, NY, USA) 1:2000 diluted in PBS-IH containing 0.5% BSA for 1 h. Cells were washed, covered with Vectashield mounting media (Vector Laboratories, Burlingame, CA, USA) and a glass coverslip, and dried overnight at 4°C. Images were taken using a Nikon A1 confocal microscope. All the steps were performed at room temperature unless mentioned otherwise.

### Flow cytometry

HEK293 stable cells and Neuro2a transiently transfected cells were treated with either 0.1% DMSO or Ipsen 5i (10^−6^ or 10^−5^ M) for 24 h at 37°C. On the day of experiment, cells were washed with ice-cold PBS-IH, detached, and precipitated by centrifugation at 500 × g for 5 min. Cells were then incubated with antibodies the same way as described above for confocal microscopy. For immunostaining of MC3R, cells were incubated with HA.11 antibody (Covance, Princeton, NJ, USA) at 1:100 dilutions and then stained with secondary antibody as described above. Cells were analyzed using a C6 Accuri Cytometer (Accuri Cytometers, Ann Arbor, MI, USA). Fluorescence of cells expressing the DMSO-treated empty vector (pcDNA3.1) was used for background staining. The expression of the mutants was calculated as percentage of DMSO-treated WT receptor expression using the following formula: [(mutant − pcDNA3.1)/(WT − pcDNA3.1) × 100%] (31).

### Intracellular cAMP accumulation assay

HEK293 stable cells and Neuro2A and N1E-115 transiently transfected cells were treated with 0.1% DMSO or different concentrations of Ipsen 5i for 24 h at 37°C. On the day of experiment, cells were washed twice with warm Waymouth’s MB752/1 media (Sigma-Aldrich, St. Louis, MO, USA) containing 1 mg/ml BSA (Waymouth’s/BSA) and incubated with Waymouth’s/BSA containing 0.5 mM isobutylmethylxanthine (Sigma-Aldrich) for 15 min. Cells were then stimulated with 10^−6^ M NDP-MSH for 1 h at 37°C. Intracellular cAMP was extracted by adding 0.5 M percholoric acid containing 180 μg/ml theophylline (Sigma-Aldrich) and neutralized with 0.72 M KOH/0.6 M KHCO_3_. Cyclic AMP concentration was determined by RIA (25). The intracellular cAMP accumulation of the mutants was calculated as percentage of DMSO-treated WT receptor using the formula [mutant/WT × 100%] for HEK293 cells or the formula [(mutant − pcDNA3.1)/(WT − pcDNA3.1) × 100%] for Neuro2a and N1E-115 cells.

### Data analysis

Data were analyzed using GraphPad Prism 4.0 software (San Diego, CA, USA). The statistical significance of the differences between DMSO and Ipsen 5i treated cells was assessed by Student’s *t*-test.

## Results

### Ipsen 5i rescued the cell surface expression of mutant MC4Rs

**Figure 1:**
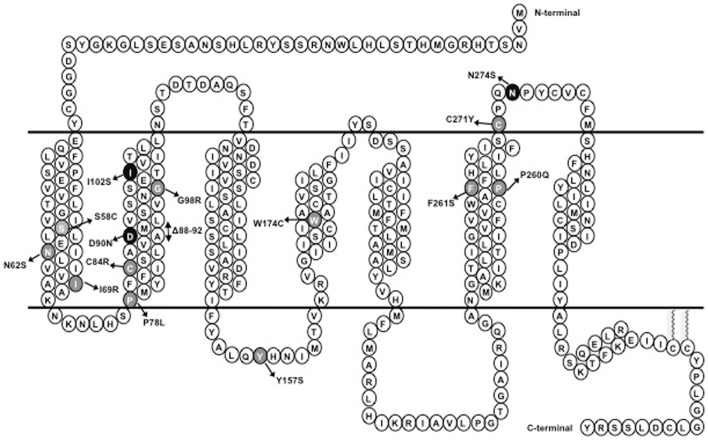
**Schematic representation of MC4R**. Naturally occurring mutations of the *MC4R* characterized in this study are highlighted; mutations that result in intracellular retention of the MC4R are highlighted in gray whereas mutations that do not interfere with the cell surface expression of the MC4R are highlighted in dark.

**Figure 2:**
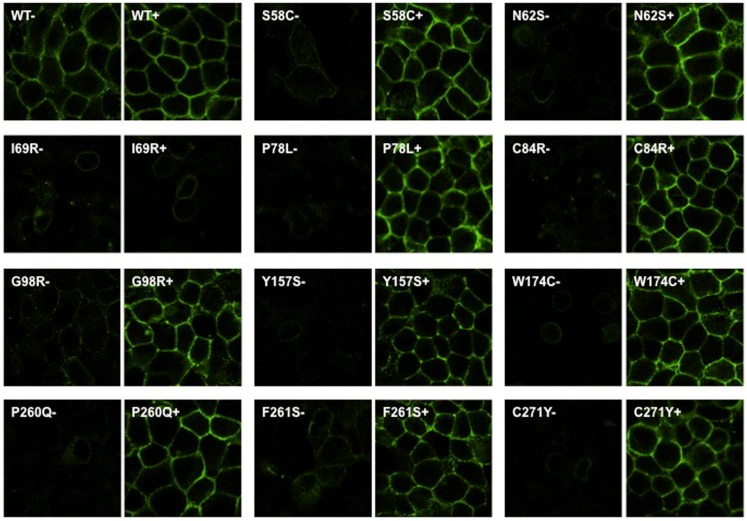
**Ipsen 5i rescued the cell surface expression of intracellularly retained mutant MC4Rs in HEK293 cells as visualized by confocal microscopy**. HEK293 cells stably expressing WT or mutant MC4Rs were treated with 10^−6^ M Ipsen 5i for 24 h and then stained with Alexa Fluor 488-conjugated antibody. Cells were visualized using a Nikon A1 confocal microscope. Results are representative of three independent experiments.

To investigate whether Ipsen 5i acted as a pharmacoperone rescuing the cell surface expression of mutant MC4Rs, we studied 11 mutants (S58C, N62S, I69R, P78L, C84R, G98R, Y157S, W174C, P260Q, F261S, and C271Y) that have reduced or absent cell surface expression (16, 28, 30) (Figure 1). We first visualized the cell surface expression of the 11 mutants stably expressed in HEK293 cells treated with 10^−6^ M Ipsen 5i using a confocal microscope. As shown in Figure 2, with 10^−6^ M Ipsen 5i treatment for 24 h, the immunostaining of 10 mutants (S58C, N62S, P78L, C84R, G98R, Y157S, W174C, P260Q, F261S, and C271Y) was dramatically enhanced compared with that of the DMSO-treated control cells whereas that of one mutant (I69R) was not obviously changed.

**Figure 3:**
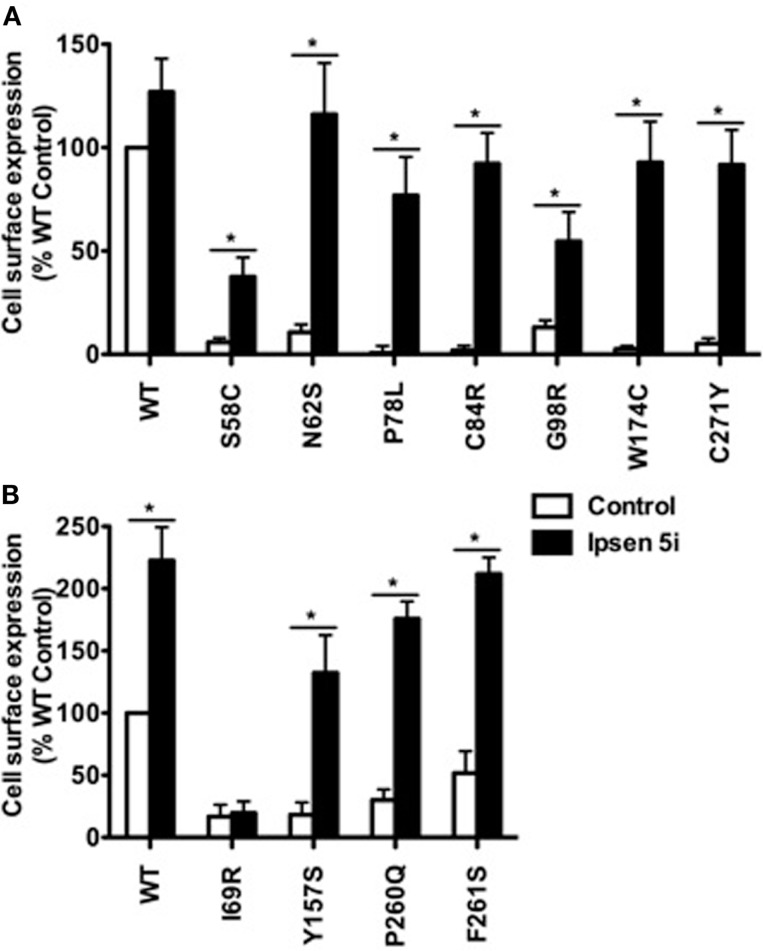
**Ipsen 5i rescued the cell surface expression of intracellularly retained mutant MC4Rs in HEK293 cells as quantitated by flow cytometry**. HEK293 cells stably expressing WT or mutant MC4Rs were treated with 10^−5^ **(A)** or 10^−6^ **(B)** M Ipsen 5i for 24 h and then stained with Alexa Fluor 488-conjugated antibody. The immunostaining was measured using a C6 Accuri Cytometer. The results are expressed as % DMSO-treated WT cell surface expression level after correction of the non-specific staining in cells expressing the empty vector. Results are shown as mean ± SEM of at least three independent experiments. *Significantly different from the DMSO-treated control group, *p* < 0.05.

To quantitate the rescuing effect of Ipsen 5i on the cell surface expression of mutant MC4Rs, flow cytometry studies were performed using HEK293 and Neuro2a cells. HEK293 cells stably expressing WT or mutant MC4Rs were treated with 10^−5^ M (Figure 3A) or 10^−6^ M (Figure 3B) Ipsen 5i for 24 h. Consistent with confocal microscopy results, the cell surface expression of 10 mutants (S58C, N62S, P78L, C84R, G98R, Y157S, W174C, P260Q, F261S, and C271Y) was significantly increased with Ipsen 5i treatment to a level similar to or even higher than that of the DMSO-treated WT receptor. I69R was not rescued by Ipsen 5i in HEK293 cells.

**Figure 4:**
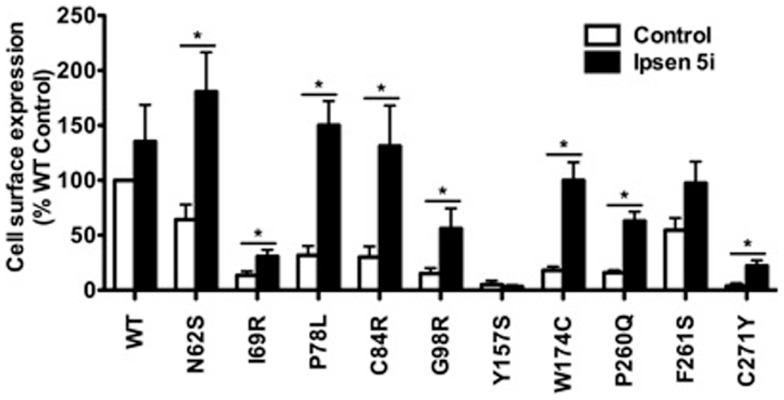
**Ipsen 5i rescued the cell surface expression of intracellularly retained mutant MC4Rs in Neuro2a cells**. Neuro2a cells transiently expressing WT or mutant MC4Rs were treated with 10^−6^ M Ipsen 5i. Results are shown as mean ± SEM of at least three independent experiments. See the legend to Figure 3 for details.

Neuro2a cells transiently expressing WT or mutant MC4Rs were treated with 10^−6^ M Ipsen 5i for 24 h and then were used for flow cytometry studies. S58C was not further studied in neuronal cells because as described later its function was not rescued by Ipsen 5i. As shown in Figure 4, the cell surface expression of eight mutants (N62S, I69R, P78L, C84R, G98R, W174C, P260Q, and C271Y) was increased with Ipsen 5i treatment compared with the DMSO-treated control group. The cell surface expression of I69R was slightly (although significantly) increased in Neuro2a cells whereas it was not increased in HEK293 cells. The increase of cell surface expression of F261S was not statistically significant in Neuro2a cells. One mutant (Y157S) that was rescued by Ipsen 5i in HEK293 cells was not rescued in Neuro2a cells.

### The majority of the mutant MC4Rs rescued with Ipsen 5i could respond to agonist stimulation with increased cAMP generation

**Figure 5:**
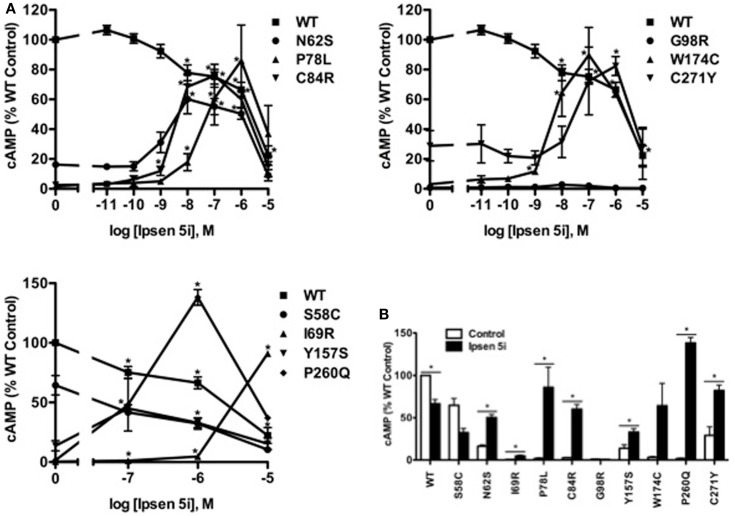
**Ipsen 5i rescued the function of intracellularly retained mutant MC4Rs in HEK293 cells**. HEK293 cells stably expressing WT or mutant MC4Rs were treated with either different concentrations of Ipsen 5i **(A)**, 10^−6^ M Ipsen 5i **(B)**, or DMSO as control for 24 h. Cells were washed twice and stimulated with 10^−6^ M NDP-MSH for 1 h. Intracellular cAMP samples were collected and cAMP concentrations were measured using RIA. The results are expressed as % DMSO-treated WT cAMP production. Data points are shown as mean ± SEM of at least two or three independent experiments. *Significantly different from the DMSO-treated control group, *p* < 0.05.

We next investigated whether Ipsen 5i-rescued mutant MC4Rs were functional in generating cAMP at the cell surface. HEK293 cells stably expressing WT or mutant MC4Rs were incubated with different concentrations of Ipsen 5i for 24 h, and then stimulated with 10^−6^ M NDP-MSH. The intracellular cAMP accumulation was measured. As shown in Figure 5, the cAMP accumulation of WT MC4R was decreased by approximately 30% with 10^−6^ Ipsen 5i treatment and by 80% with 10^−5^ M Ipsen 5i treatment. We observed an increase in cAMP accumulation at 10^−9^ M Ipsen 5i for C84R and W174C and a maximal increase at a concentration between 10^−8^ and 10^−6^ M for N62S, P78L, C84R, Y15YS, W174C, P260Q, and C271Y. Unlike most of the mutants that decreased cAMP accumulation at 10^−5^ M Ipsen 5i, I69R had a maximal cAMP accumulation at that concentration. The signaling of S58C and G98R was not increased by Ipsen 5i.

**Figure 6:**
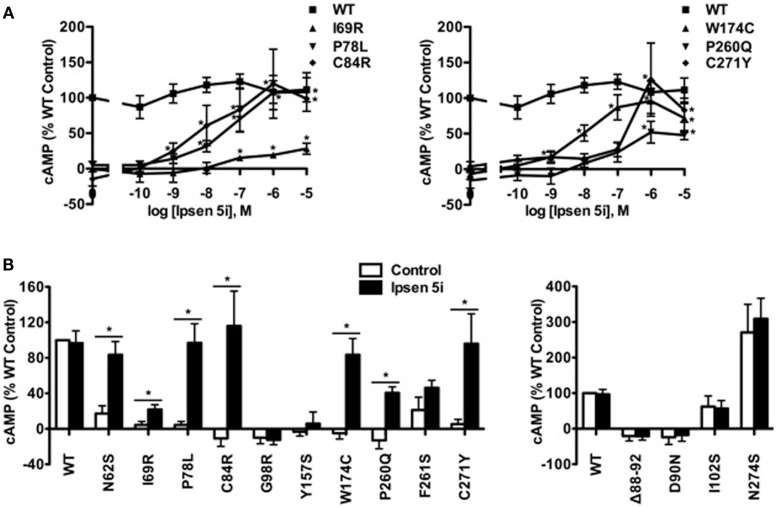
**Ipsen 5i rescued the function of intracellularly retained mutant MC4Rs in Neuro2a cells**. Neuro2a cells transiently expressing WT or mutant MC4Rs were treated with either different concentrations of Ipsen 5i **(A)**, 10^−6^ M Ipsen 5i **(B)**, or DMSO as control. The results are expressed as % DMSO-treated WT cAMP production after correction of the cAMP production in cells expressing the empty vector. Data points are mean ± SEM of at least three independent experiments. See the legend to Figure 5 for other details.

**Figure 7:**
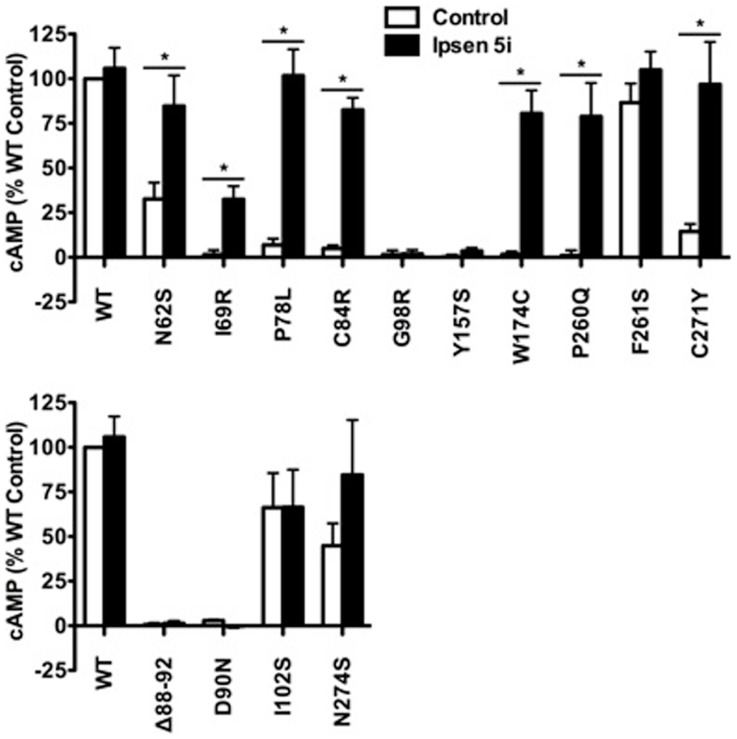
**Ipsen 5i rescued the function of intracellularly retained mutant MC4Rs in N1E-115 cells**. N1E-115 cells transiently expressing WT or mutant MC4Rs were treated with 10^−6^ M Ipsen 5i or DMSO as control. The results are expressed as % DMSO-treated WT cAMP production after correction of the cAMP production in cells expressing the empty vector. Results are shown as mean ± SEM of at least three independent experiments. See the legend to Figure 5 for details.

In Neuro2a cells transiently expressing MC4Rs, we also observed an increase in cAMP accumulation at 10^−9^ M Ipsen 5i and a maximal increase at 10^−6^ M Ipsen 5i (or at 10^−5^ M Ipsen 5i for I69R) in Neuro2a cells (Figure 6A). As shown in Figure 6B, with 10^−6^ M Ipsen 5i treatment in Neuro2a cells, seven mutants (N62S, I69R, P78L, C84R, W174C, P260Q, and C271Y) had significantly increased cAMP accumulation whereas signaling of three mutants (G98R, Y157S, and F261S) was not increased by Ipsen 5i. High concentration of Ipsen 5i did not decrease the cAMP accumulation of WT or mutant MC4Rs as dramatically as seen in HEK293 cells (Figure 6A). Our results obtained from N1E-115 cells were similar with those obtained from Neuro2a cells (Figure 7).

### Ipsen 5i did not affect mutant MC4Rs that are expressed at the cell surface

To investigate whether Ipsen 5i rescued the function of mutant MC4Rs that are expressed at the cell surface but have other defects, we studied four mutants that are defective in ligand binding [(Δ88-92) (32) (Class III according the classification proposed by Tao (33)], signaling (D90N and I102S) (29, 34) (Class IV), or with no obvious defect (N274S) (28) (Class V). As shown in Figures 6B and 7, 10^−6^ Ipsen 5i had no effect on the signaling of these four mutants.

### Specificity of the MC4R mutant rescue

**Figure 8:**
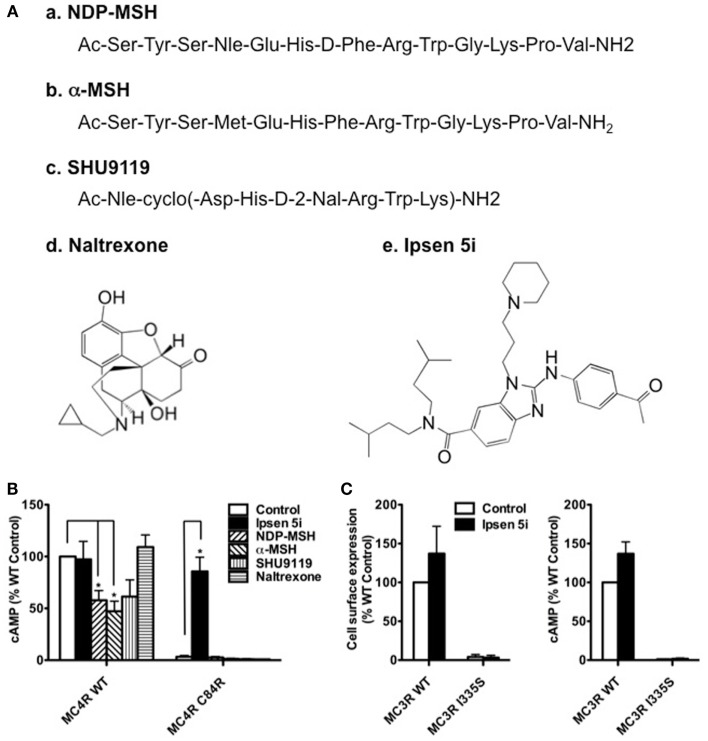
**Ipsen 5i specifically rescued mutant MC4Rs and the rescue action occurred intracellularly**. **(A)** Structures of the ligands studied. The peptide ligands NDP-MSH and α-MSH are agonists of the MC4R whereas SHU9119 is an MC4R antagonist. Naltrexone is a small molecule antagonist of the opioid receptor. Ipsen 5i is a small molecule inverse agonist of the MC4R. **(B)** Neuro2a cells transiently expressing WT or C84R MC4R were treated with different ligands for 24 h (10^−5^ M NDP-MSH, α-MSH, SHU9119 or naltrexone or 10^−6^ M Ipsen 5i). Cells were washed twice and then stimulated with 10^−6^ M NDP-MSH for 1 h and intracellular cAMP concentrations were measured. **(C)** Neuro2a cells transiently expressing WT or I335S MC3Rs were treated with 10^−6^ M Ipsen 5i for 24 h. Then, the cell surface expression and intracellular cAMP production of MC3Rs were measured. Results are shown as mean ± SEM of at least three independent experiments. *Significantly different from the DMSO-treated control group, *p* < 0.05.

To investigate whether cell impermeable peptide ligands of the MC4R or cell permeable ligands of other receptors could rescue mutant MC4Rs, we studied the effect of two MC4R peptide agonists (NDP-MSH and α-MSH), one MC4R peptide antagonist (SHU9119), and one pharmacoperone of δ opioid receptor (naltrexone) (35) (Figure 8A) on C84R MC4R. As shown in Figure 8B, NDP-MSH and α-MSH decreased the signaling of WT MC4R by approximately 50%. However, none of the four ligands rescued the signaling of C84R MC4R.

To investigate whether Ipsen 5i specifically rescues mutant MC4Rs, we studied the effect of Ipsen 5i on one of intracellularly retained mutant MC3Rs (I335S) (27, 36). As shown in Figure 8C, Ipsen 5i had no effect on the cell surface expression or signaling of I335S MC3R.

## Discussion

Most of the inactivating mutations in GPCRs causing human diseases result from protein misfolding and subsequent retention and degradation by the ER quality control system (22). Misrouted receptors may retain intrinsic function and become functional when correctly located (37). Pharmacoperones that can permeate plasma membrane specifically stabilize the conformation and correct the trafficking of misfolded receptors, thus, rescuing the receptor and curing human diseases (38–41). In the current study, we identified Ipsen 5i, an antagonist of the MC4R, as a potent pharmacoperone specifically rescuing the cell surface expression and function of intracellularly retained mutant MC4Rs.

Our results showed that all 11 intracellularly retained mutants studied herein could be rescued to the cell surface by Ipsen 5i in at least one cell line (Table 1). Y157S could be significantly rescued in HEK293 cells but not in neuronal cells whereas I69R was partially rescued in neuronal cells but not in HEK293 cells. The effects of Ipsen 5i on eight mutants were similar between HEK293 and neuronal cell lines. The cell surface expression of most mutants treated with Ipsen 5i was increased to at least 50% of or even similar to that of the DMSO-treated WT receptor. The rescuing efficacies of Ipsen 5i were different for different mutations. I69R was the most difficult to rescue because it could only be maximally rescued with the highest concentration of Ipsen 5i (10^−5^ M) and could not be rescued with another pharmacoperone of the MC4R, ML00253764 (unpublished observations). This suggests that I69R induces a large change in the receptor conformation that is difficult to be stabilized.

**Table 1:** **Summary of the effects of Ipsen 5i on the cell surface expression and function of WT and mutant MC4Rs**.

Mutants	HEK293 cells		Neuronal cells	
	Expression	Function	Expression	Function
WT	↑	↓	−	−
S58C	↑	−	/	/
N62S	↑	↑	↑	↑
I69R	−	↑	↑	↑
P78L	↑	↑	↑	↑
C84R	↑	↑	↑	↑
G98R	↑	−	↑	−
Y157S	↑	↑	−	−
W174C	↑	↑	↑	↑
P260Q	↑	↑	↑	↑
F261S	↑	/	−	−
C271Y	↑	↑	↑	↑

*↑, increase; ↓, decrease; −, no significant effect; /, not studied herein*.

Eight of the 11 mutants rescued to the cell surface were functional in cAMP production (N62S, I69R, P78L, C84R, Y157S, W174C, P260Q, and C271Y) in HEK293 cells and seven mutants, with the exception of Y157S, in neuronal cells (Table 1). These results suggest that these mutants, although misfolded and retained intracellularly, retain the ability to bind to agonist and initiate downstream signaling. Ipsen 5i treatment did not significantly increase F261S signaling. G98R, although rescued to the cell surface, did not respond to NDP-MSH stimulation with increased cAMP generation, suggesting that this mutant was also defective in ligand binding and/or signaling. We are also unable to rescue G98R functionally using a small molecule agonist as the pharmacoperone (42). Despite low cell surface expression, S58C has significant signaling (28), likely due to the presence of spare receptor (33). Ipsen 5i treatment significantly increased cell surface expression of S58C (Figures 2 and 3). However, its signaling was not increased and tended to decrease.

The signaling of WT MC4R was dramatically decreased when treated with 10^−5^ M Ipsen 5i in HEK293 cells (Figure 5). The residual Ipsen 5i that had not been washed away presumably still occupied the binding site of the MC4R and therefore, antagonized the stimulation of NDP-MSH. Although Ipsen 5i has a high-affinity with the MC4R (*K_i_*, 2 nM), it has relatively low functional antagonist potency (77 nM) (24), minimizing its antagonizing effect on NDP-MSH. Indeed, in our study, 10^−6^ and 10^−7^ M Ipsen 5i, which already had significant pharmacoperone rescuing ability, only decreased the signaling of WT MC4R by approximately 30 or 20% in HEK293 cells, respectively (Figure 5). Interestingly, we had not observed such dramatic decrease in WT MC4R signaling in neuronal cells (Figures 6 and 7), suggesting that it might be easier for Ipsen 5i to dissociate from the MC4R expressed in neuronal cells.

Ipsen 5i has low affinity for the MC3R (*K_i_*, 400 nM) and therefore, we investigated whether Ipsen 5i could rescue misrouted MC3R mutant I335S. We found that Ipsen 5i did not increase the cell surface expression or function of I335S MC3R, suggesting that Ipsen 5i was a pharmacoperone specific for the MC4R. Although Ipsen 5i was a potent pharmacoperone of the MC4R, it had no effect on mutant MC4Rs defective in ligand binding or signaling, suggesting that Ipsen 5i could only rescue the function of the intracellularly retained mutant MC4Rs. As expected, cell impermeable peptide ligands of the MC4R did not rescue the function of misrouted mutant MC4R C84R whereas cell permeable Ipsen 5i did. Consistent with previous reports on several other GPCRs (35, 43–45), this observation suggests that only cell permeable small compound could act as a pharmacoperone and the rescuing action occurred intracellularly. Peptide ligands decreased the signaling of WT MC4R probably by inducing internalization and down-regulation (NDP-MSH and α-MSH) or by antagonizing NDP-MSH (SHU9119). Naltrexone, a pharmacoperone of δ opioid receptor (35), also did not correct the function of C84R MC4R, suggesting that only ligands for the MC4R could act as MC4R pharmacoperones.

In summary, Ipsen 5i increased the cell surface expression of all 11 intracellularly retained mutant MC4Rs (100%) studied herein and eight of the 11 mutants (73%) were functional at the cell surface in at least one cell line. Ipsen 5i could rescue mutant MC4Rs at a concentration as low as 10^−9^ M. To our knowledge, it was the most potent pharmacoperone of the MC4R identified so far. Future experiments aimed at demonstrating the *in vivo* efficacy of this ligand in transgenic animals will represent another important step toward personalized medicine for treating patients harboring these MC4R mutations.

## Conflict of Interest Statement

The authors declare that the research was conducted in the absence of any commercial or financial relationships that could be construed as a potential conflict of interest.

## Abbreviations

DMSO, dimethyl sulfoxide; ER, endoplasmic reticulum; GPCR, G protein-coupled receptor; HEK, human embryonic kidney; MC3R, melanocortin-3 receptor; MC4R, melanocortin-4 receptor; MSH, melanocyte stimulating hormone; WT, wild type.
